# Supplementary material for: Optimization of Novel Human Acellular Dermal Dressing Sterilization for Routine Use in Clinical Practice
Source: Int J Mol Sci. 2021 Aug 6;22(16):8467. doi: 10.3390/ijms22168467 (PMC8395076; doi:10.3390/ijms22168467)
Supplement: Supplementary file 1 [file ijms-22-08467-s001.zip › ijms-1280664-supplementary.pdf]

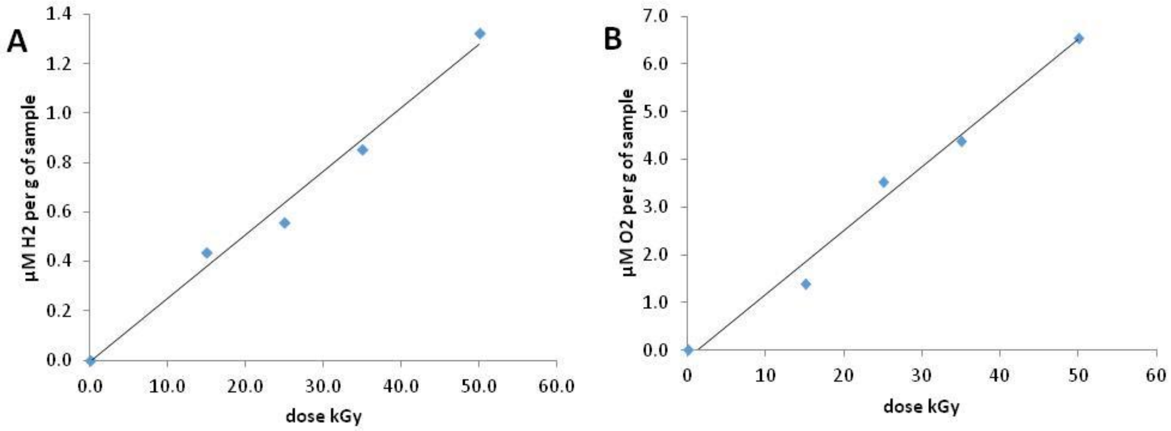

Figure S1. Dose-dependence of H_2 production (A) and O_2 consumption (B) in irradiated collagen, measured by gas chromatography. The yields: 0.026 μM H_2/J, and 0.12 μM O_2/J.
